# Anthropogenic Food Subsidy to a Commensal Carnivore: The Value and Supply of Human Faeces in the Diet of Free-Ranging Dogs

**DOI:** 10.3390/ani8050067

**Published:** 2018-04-27

**Authors:** James R. A. Butler, Wendy Y. Brown, Johan T. du Toit

**Affiliations:** 1 CSIRO Land and Water, GPO Box 2583, Brisbane, QLD 4001, Australia; 2 Canine and Equine Research Group, University of New England, Armidale, NSW 2351, Australia; wbrown@une.edu.au; 3 Department of Wildland Resources, Utah State University, 5230 Old Main Hill, Logan, UT 84322-5230, USA; johan.dutoit@usu.edu

**Keywords:** canine nutrition, coprophagy, Dog Development Index, rabies, scavenging, social ecology

## Abstract

**Simple Summary:**

Free-ranging dog populations are growing worldwide, posing threats to human health and wildlife conservation. Dog population management requires a better understanding of the supply and quality of human-derived food. We studied the diet of free-ranging dogs in a remote area of Zimbabwe to explore the relationships between human waste, dog condition, and fertility, with a focus on the value of human faeces. We found that mammal remains, ‘sadza’ (maize porridge, the human staple) and human faeces were the most important food items, and unlike other items, faeces and sadza were consistently available. Nutritional analysis showed that human faeces was comparatively high in protein and energy content, exceeding that of sadza, and equivalent to mammal remains. Adult female dog condition was good throughout the year, indicating a diet sufficient to maintain fertility, and dogs largely fed alone, suggesting that food was abundant. We conclude that the lack of sanitation allows an important food subsidy to free-ranging dogs, but improved sanitation is unlikely to limit population growth as long as alternative human waste is available. Reproductive control by owners is more likely to reduce fertility rates.

**Abstract:**

As the global population of free-ranging domestic dogs grows, there is increasing concern about impacts on human health and wildlife conservation. Effective management of dog populations requires reliable information on their diet, feeding behavior, and social ecology. Free-ranging dogs are reliant on humans, but anthropogenic food subsidies, particularly human faeces (i.e., coprophagy) have not previously been fully quantified. In this study we assess the contributions of different food types to the diet, and their influences on the social behaviour of free-ranging dogs in communal lands of rural Zimbabwe, with a focus on coprophagy. Free-ranging dog diets, body condition, and sociology were studied amongst 72 dogs over 18 months using scat analysis and direct observations. Human faeces constituted the fourth most common item in scats (56% occurrence) and contributed 21% by mass to the observed diet. Human faeces represented a valuable resource because relative to other food items it was consistently available, and of higher nutritional value than ‘sadza’ (maize porridge, the human staple and primary human-derived food), yielding 18.7% crude protein and 18.7 KJ/kg gross energy, compared to 8.3% and 18.5 KJ/kg for sadza, respectively. Human faeces had protein and energy values equivalent to mammal remains, another important food item. Dog condition was generally good, with 64% of adult females and 74% of adult males in the highest two body condition scores (on a five point scale), suggesting a plentiful and high quality food supply. Dogs largely fed alone, perhaps as a consequence of the small, inert, and spatially dispersed items that comprise their diet, and its abundance. We discuss the relationships between sanitation, human development, the supply of human faeces, female dog fertility, and population control.

## 1. Introduction

The global population of domestic dogs *Canis familiaris* has been conservatively estimated to be 700 million and growing [1]. Most animals are owned, and in countries or regions with lower levels of socio-economic development, many owned dogs are free-ranging. This reflects a lower ‘Dog Development Index’, which is characterized by limited dog husbandry and investment in their welfare due to owners’ lack of resources and capacity [2]. Free-ranging dogs pose a threat to human health as vectors of zoonoses, most notably rabies [3], and other pathogens [4]. They also threaten wildlife conservation [5] as competitors with other carnivores for resources [6,7], and as reservoirs and vectors of disease [8], particularly if preyed upon by wild carnivores [2,9]. The understanding of dog–human relationships and demographics is important for improved management [10], and this is most essential in remote and less developed regions where these problems are most prevalent [11].

Crucial to the persistence of free-ranging dog populations commensal with humans is the availability of anthropogenic food [5]. Dogs are efficient scavengers of waste, frequently outcompeting wild species [7], and even specialists such as vultures [6,12]. In rural areas of less developed countries, free-ranging dogs subsist on waste in and around villages, including coprophagy of human faeces [6,13,14]. It has been suggested that as a result of the dispersed and inert nature of this food, free-ranging dogs have little incentive to form cooperative groups [15,16]. The nature of human waste may have also played a role in the domestication of wolves *Canis lupus*; the ‘self-domestication hypothesis’ proposes that early proto-dogs abandoned the conspecific group hunting of wolves due to the alternative availability of inert human waste [17]. 

This paper is part of a broader study of the ecology, demography, rabies epidemiology, and wildlife interactions of free-ranging dogs in Zimbabwe’s rural communal lands [2,6,7,8,9,18,19,20]. Communal lands cover 42% of the country’s land area and are broadly representative of many rural areas in sub-Saharan Africa in terms of dog ecology and dog–human relationships [19]. In 1990 it was estimated that 71% of Zimbabwe’s dog population lived in communal lands [21], but this proportion is now likely to be higher due to rapid population growth [19]. A key question is how the free-ranging dog population continues to grow despite high mortality rates. One untested hypothesis is that a consistent supply of anthropogenic food subsidies enables adult females to reproduce successfully throughout the year, offsetting high mortality [19].

The objective of this study was to analyse the diet of free-ranging dogs in a remote area in which the livelihoods of the human population are based on traditional small-scale agriculture. We assessed the supply of food to free-ranging dogs, paying particular attention to the previously unquantified dietary contribution of human faeces. We also assessed possible dietary effects on dog condition and social ecology. Finally, we considered the implications of our results for the management of dog populations in regions where the Dog Development Index is low.

## 2. Materials and Methods

### 2.1. Study Area

**Figure 1:**
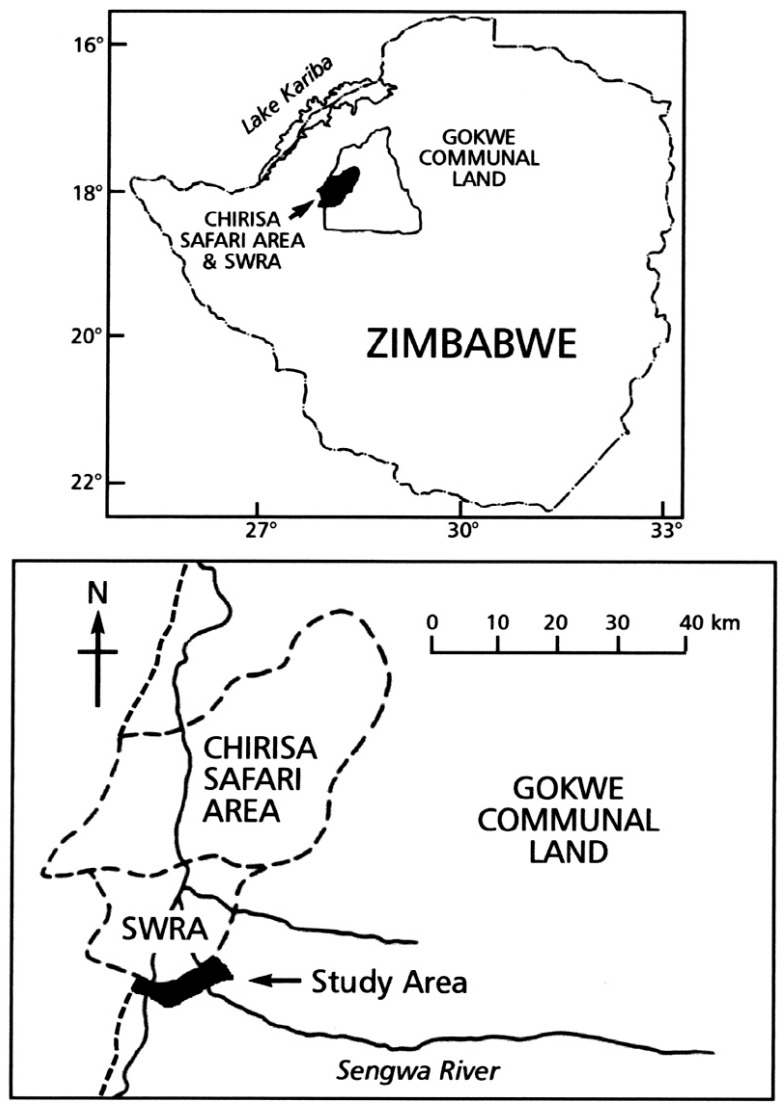
The location of the Gokwe Communal Land study area and the Sengwa Wildlife Research Area (SWRA) in northwestern Zimbabwe.

The study was conducted between January 1995 and June 1996 in a 33-km^2^ section of Gokwe Communal Land (GCL) bordering (for 16 km) the Sengwa Wildlife Research Area (SWRA), which adjoins the Chirisa Safari Area in northwestern Zimbabwe (Figure 1). The climate is characterised by a wet season (November–April) and dry season (May–October). Mean annual rainfall and temperature is 662 mm and 22.2 °C, respectively. Dominant vegetation types are *Brachystegia-Julbernardia* and *Colophospermum* mopane savanna woodlands. The GCL-SWRA boundary is fenced, but domestic and wild animals traverse through or over it, so these species co-exist [6,9,20].

### 2.2. Livelihoods and Waste Management

In October 1995 (mid-way through the study), a questionnaire survey was carried out which covered all 130 households within the study area. The survey design mirrored a national survey of dog–human relationships carried out in 1994, which included the GCL, and asked households about their family size and ages, waste disposal methods, livestock, reasons for dog ownership, dogs’ ages and sexes, life histories, and husbandry [19].

**Figure 2:**
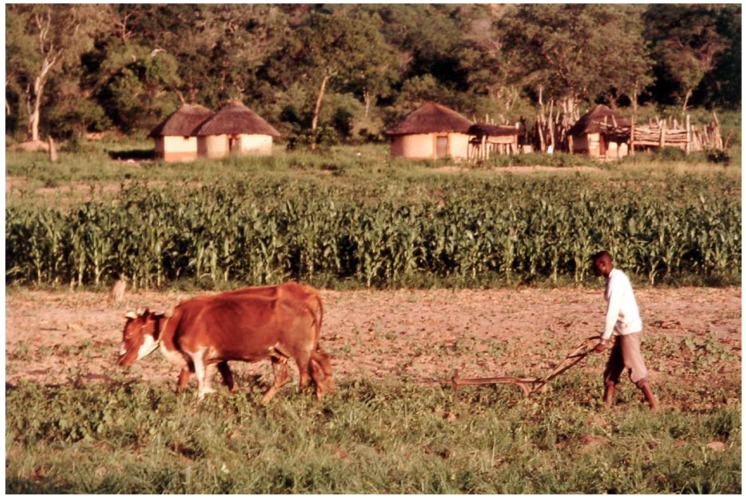
A typical homestead and livelihood activity in the study area (photo: James Butler).

The survey recorded a population of 937 people in the study area. Homesteads were typical of communal lands, consisting of thatched huts within an unfenced yard. Livelihoods were based on traditional agro-pastoralism, with maize, sorghum, millet, and cotton grown in fields around homesteads, and cattle *Bos indicus*, goats *Capra hircus*, donkeys *Equus asinus*, and sheep *Ovis aries* grazed on natural vegetation and crop residues (Figure 2). Animals were enclosed in a protective ‘kraal’ at night to prevent predation by wild carnivores. Most households owned poultry (largely chickens *Gallus domesticus*), and 19% owned at least one domestic cat *Felis catus.*

Leftover food and other domestic waste was disposed of in pits on the household perimeter. Disease or predation, the main causes of livestock mortalities, generated carcasses of which 59% were left in situ, and the rest were burned, buried, or eaten by their owners. Wildlife carcasses also occurred in the SWRA and GCL, either because of predation by wild carnivores, snaring by humans, or disease. During the study a total of 199 carcasses were available for consumption by scavengers in the GCL study area, with a combined biomass of 22,957 kg and density of 696 kg/km^2^. In the adjacent 1 km-deep strip of the SWRA, 23 carcasses were available in the same period, with a biomass of 7738 kg and density of 484 kg/km^2^ [6].

The survey also recorded that 120 (92%) of households did not possess a latrine or toilet, and members of these households usually defaecated in the open. Studies in other communal lands have calculated that adults produce on average 0.16 kg of faeces/24 h, and children (<17 years old) 0.13 kg/24 h [22]. There was a total of 349 adults and 499 children in the 120 households without latrines; consequently each household produced on average 1 kg faeces/24 h. Over the study’s 18 months this equated to a total potential biomass of 64,800 kg in the GCL study area, with a density of 1964 kg/km^2^.

### 2.3. Dog Population and Ecology

The survey recorded a population of 236 dogs owned by 83 households. As in other communal lands [19], there was no evidence of a feral (i.e., unowned) dog population. The dog population’s structure was skewed towards juveniles, with 61% aged ≤1 year and only 11% aged >3 years, indicating a high rate of mortality. The primary cause of death was unidentified disease, followed by ‘unknown causes’ and predation by wild carnivores [9,19]. Dogs were kept primarily to protect the household, to deter wildlife from crops and wildlife, and for hunting [19]. All dogs were unrestricted and were allowed to roam freely. Radio-telemetry of focal animals (see Section 2.4.1 below) demonstrated that their home ranges included other homesteads (including dog-owning houses) and the periphery of the SWRA [2]. Dogs received no formal veterinary treatment, but there were three government rabies vaccination campaigns during the study. Rabies is endemic in Zimbabwean communal lands, and cases were recorded throughout the study amongst dogs and other livestock [8,9]. Distemper and parvovirus were also evident amongst dogs [9].

### 2.4. Methods

#### 2.4.1. Dietary Analysis

Dietary data were collected from the analysis of dog faeces (scats) collected monthly from 16 adult (>1 year old) animals (eight males and eight females) owned by 10 households distributed roughly equidistant from one another across the study area. These households were selected because they did not own latrines or toilets and were therefore typical of the vast majority in the study area. These dogs are termed ‘focal animals’ and were studied continually until they died or disappeared. Adults were chosen in order to closely monitor their diet relative to their body condition and potential fertility (see Section 2.4.3 below).

Scats were also collected monthly from 56 other adult and juvenile (≤1 year) dogs owned by the focal animals’ households or by neighbouring households, totalling a sample of 72 dogs. Scats were soaked in 10% buffered formaldehyde, drained through a 2 mm mesh sieve and broken up for analysis. Food remains were identified with a low-power (×10) microscope from a reference collection of possible food collected in the study area and adjacent SWRA. The total and monthly percentage frequency of occurrence of each food type was calculated for all faeces collected from the focal animals plus the 56 other dogs.

Identifying human faeces in scat analysis is problematic, because many of the food types eaten by humans are also eaten by dogs [13,14]. The staple human food is ‘sadza’, a porridge usually made from maize, and this is fed to dogs (see Section 3 Results). Thus, a dog leaving a scat containing sadza may have either been fed sadza, eaten leftover sadza, or consumed human faeces containing undigested sadza. People usually eat sadza accompanied by relish made from cooked brassicas, tomatoes, and onions, and so remains of relish and sadza together in scats indicated consumption of human faeces. However, if they occurred alone it was assumed that the dog had eaten sadza or vegetables directly. The presence of toilet paper was also indicative of coprophagy.

Over-representation of small prey complicates the use of scat analysis and frequency of occurrence to quantify carnivore diets [23,24,25]. Dogs are scavengers that are also capable of predation, making it impossible to deduce from scats the proportions of the diet derived from scavenging or predation. Other studies have redressed this issue by augmenting scat analysis with direct observations of feeding e.g., [23]. We adopted this approach to quantify the contribution of human faeces to the diet, and to determine the proportion of the diet that was an anthropogenic subsidy.

Focal animal observations extended over multiple 3-, 6-, or 12-h sessions arranged to cover the entire 24-h diurnal-nocturnal cycle for each animal. The 16 focal animals were observed for a total of 486 h, with an average of 30.4 h/animal; 75% of the observation time was during daylight and 25% was at night. Two observers carried out the focal animal observations, one to observe and one to record. A following distance of ≤20 m behind the focal animal minimized disturbance and enabled identification of food items using binoculars and/or a handheld flashlight. The mass eaten at each separate feeding observation (‘meal’) was estimated visually, and regularly calibrated by measuring the wet mass of the same food types in the laboratory. Whether the meal was deliberately fed by people or gained independently was also recorded.

#### 2.4.2. Nutritional Value of Food Items

The nitrogen content of human faeces and sadza was determined using a nitrogen analyser, from which crude protein concentration was calculated (where crude protein = N × 6.25), and a bomb calorimeter was used to calculate gross energy (MJ/kg). Samples of fresh human faeces and sadza were collected from the study area, dried to a constant mass, and five sub-samples of 100 g were tested for each food type. Results were compared with data from the literature in Zimbabwe for other food items in the dogs’ diets.

#### 2.4.3. Dog Condition

A qualitative condition score was awarded monthly to every focal animal and other associated adult animals sighted in July 1995 to June 1996. The score used a five-point scale based on the visibility of skeletal features under the skin [26], where 5 represented very good condition and 1 represented very poor (see Supplementary Material). Through their names, characteristic pelage colourations, ages, and sex, every focal and other dog could be individually recognized. Each individual was scored once per month and the data were analysed separately for females and males, and then aggregated. Frequencies of occurrence of females and males in each condition category were compared by χ^2^ test.

#### 2.4.4. Sociology and Diet

The duration of all observed meals was recorded in minutes. The numbers of other dogs feeding with the focal animal on the same food item was also recorded.

#### 2.4.5. Free and Prior Informed Consent

The research methodology was reviewed and endorsed by the University of Zimbabwe’s Research Board and the Zimbabwe Department of National Parks and Wildlife Management, who issued a permit for the project to be carried out in the SWRA. All focal animals’ owners were asked for their consent to study their dogs. The project’s aims and activities were explained to each household, and its neighbours. Prior to collecting scats or observing focal animals, the owners’ permission was sought 3–4 days in advance. If the household wished to withdraw from the study they were informed that they could do so at any point, but none did. If a focal animal died or disappeared, permission was sought to recruit another adult dog into the study from the same household.

## 3. Results

### 3.1. Diet

#### 3.1.1. Scat Analysis

The analysis of 945 dog scats indicated a wide range of food (Table 1). The four most frequently occurring items were (in descending order) sadza (87.9% of scats), mammal remains (81.3%), vegetables and fruit (69.8%), and human faeces (56.2%). Twenty-four mammal species were identified, 18 of which were wild species, and six domestic (including dog). The three most common species were goat, springhare *Pedetes capensis* and cow; the remaining 21 species occurred rarely. Of note was the occurrence of eight carnivores, six of which were wild species. Larvae of the hawkmoth *Herse ipomoea* and winged termites *Macrotermes* spp. constituted the bulk of insects eaten. The only identified bird eaten was chicken.

**Figure 3:**
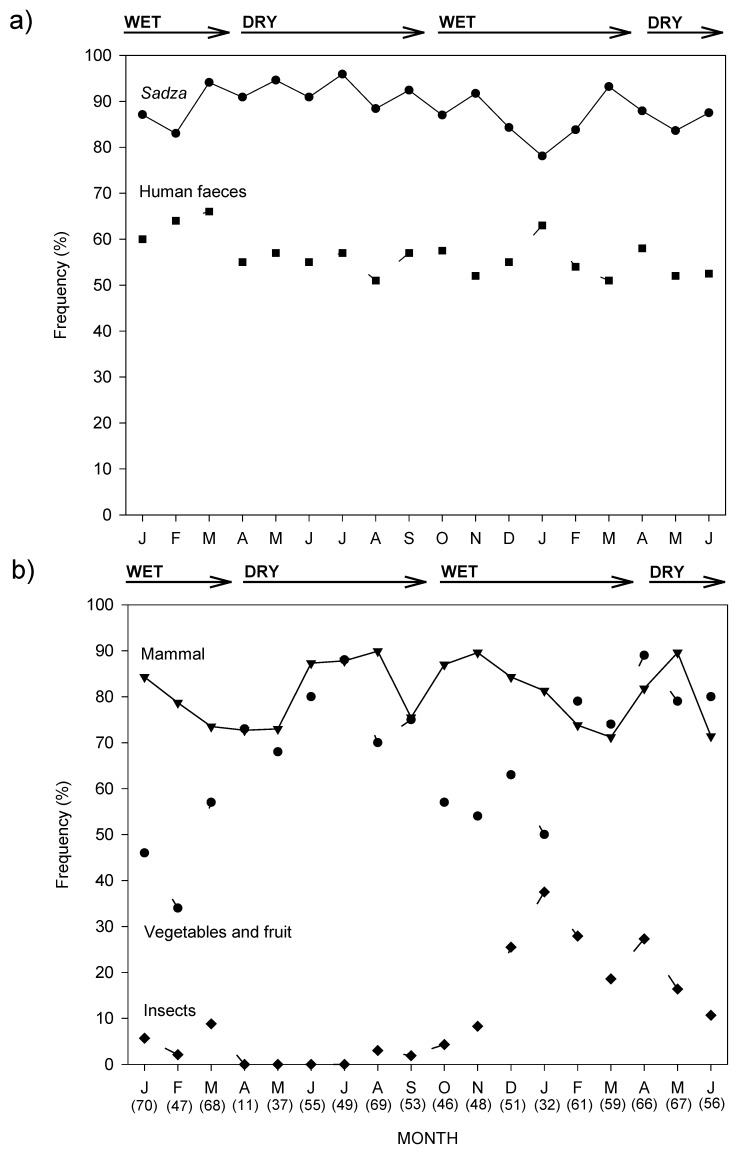
Monthly (January 1995–June 1996) percentage frequency of occurrence in dog scats of (**a**) sadza and human faeces, and (**b**) mammalian remains, vegetables and fruit, and insects, through dry and wet seasons. Numbers in brackets indicate the monthly sample size of scats analysed.

The monthly frequency of occurrence of human faeces and sadza showed no marked seasonal pattern (Figure 3a). Mammal remains occurred more erratically and did not show a seasonal pattern (Figure 3b). By comparison, the occurrence of vegetables (e.g., pumpkin and groundnuts) and fruits (e.g., cultivated watermelon and wild fruits) were seasonal, with an increase in frequency in the late wet season and early dry season (April–August), coinciding with fruiting and harvesting months. Insects were also highly seasonal, peaking during the wet season (Figure 3b).

#### 3.1.2. Observed Diet

A total of 689 meals was recorded during the 486 h of observation, with a total mass of 45.7 kg (Table 1). As indicated by scat analysis, the observed diet was wide-ranging. However, only 10 of the 24 mammal species found in scats were observed being eaten: five wild and five domestic. As a proportion of mass ingested, the three most important food items recorded were mammal meat, bones, and skin (48.8%), sadza (22.1%), and human faeces (20.5%). Other than insects, all food items eaten by dogs were inert, and none were killed independently by the dog. Vegetables and fruit, grass, insects, and chicken were far less important in the observed diet than indicated by their frequency of occurrence in scats (Table 1). Dogs also ate small amounts of baboon, ungulate, cat, and chicken faeces. 

**Figure 4:**
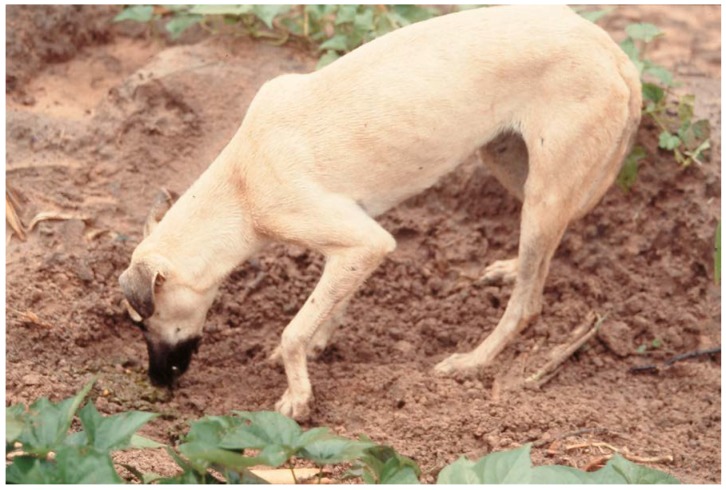
A free-ranging dog uncovering and eating human faeces buried in a field. This animal represents condition score 4 (photo: James Butler).

Human-derived food (i.e., sadza, cultivated vegetables and fruits, domestic mammals and poultry, and human faeces) contributed 88% of the mass eaten. Dogs found only 28% of their food within homesteads, and the balance came from the hinterland around homesteads. This was the case for all human faeces and carcasses. A dog would have several homesteads within its home range which it would visit while foraging. Having inspected its own home, a dog would travel directly to the next, where the yard and perimeter of the house was searched for leftover food, and also the pit and kraal on the perimeter. Human faeces was located by smell, and if buried would be dug up (Figure 4). This pattern was repeated at each homestead.

Most (87%) of the diet was independently-gained, and 13% was deliberately fed by people. The fed diet consisted almost entirely of sadza. The independent diet was largely scavenged domestic and wild mammal carrion (55.1%), of which the majority came from carcasses, and the remainder from slaughter offal. Human faeces contributed 23.6% of the independent diet, and leftover sadza contributed 10.6%.

### 3.2. Nutritional Value of Food Items

The mean (±SD) crude protein content for human faeces was more than double that for sadza (18.7% ±1.55 versus 8.3% ±0.03). The protein value of human faeces was comparable to the lower range of crude protein of mammal tissue in human diets in Zimbabwe sampled by Chitsiku [27], the mid-range for chicken, vegetables and fruit, but exceeded the value for insects (Table 2).

The mean (±SD) gross energy content of human faeces (18.7 MJ/kg ±0.01) slightly exceeded that of sadza (18.3 MJ/kg ±0.21). Faeces was comparable to the upper range of energy contents for mammal tissue, vegetables and fruit, but exceeded the content of insects and chicken (Table 2).

### 3.3. Dog Condition

**Figure 5:**
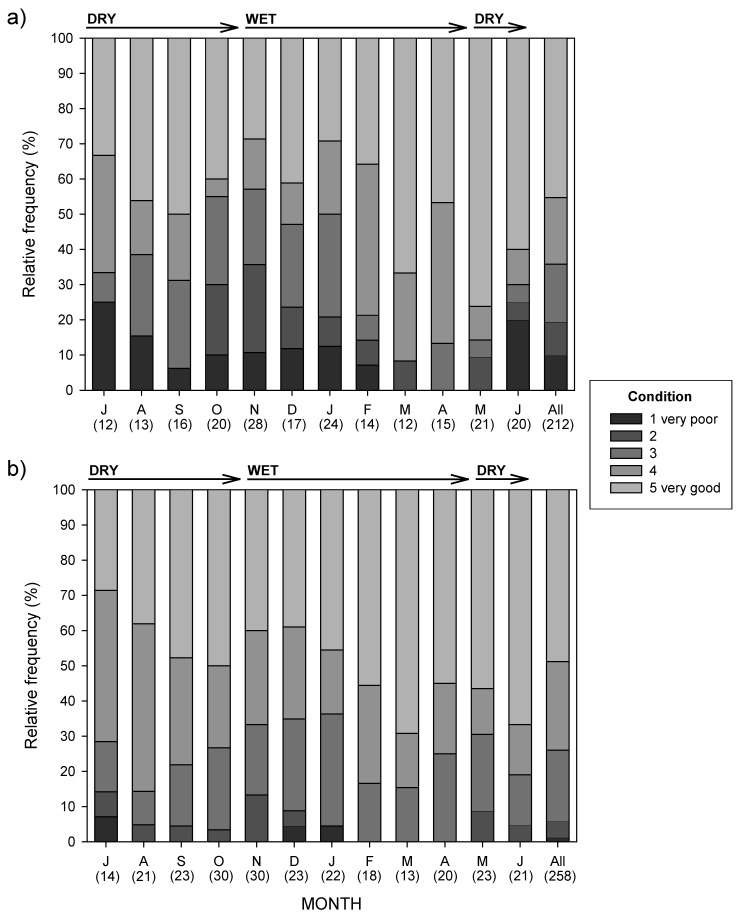
Relative frequency of monthly (July 1995–June 1996) and overall condition scores for (**a**) adult female and (**b**) adult male dogs, through dry and wet seasons. Numbers in brackets indicate the monthly or total sample sizes of records.

For adult females, in every month the greatest proportion of dogs were in condition score 5 (i.e., very good), except in February when the greatest proportion was condition 4 (Figure 5a; see also Figure 4). There was no seasonal pattern to condition scores. Overall, 64.2% of records were condition score 5 or 4.

For adult males the greatest proportion of dogs sighted had a condition score of 5 except in July and August, when the highest proportion was in condition 4 (Figure 5b). There also appeared to be no seasonal pattern to condition scores. Overall, 74.0% of records were condition 5 or 4. Adult males seemed to be in slightly better monthly condition than females, with fewer in condition 1 (1.2% versus 9.9%) and 2 (4.6% versus 9.4%); however, the differences in the relative occurrence of overall condition scores were not statistically significant (χ^2^ = 9.90, df = 4, *p* > 0.05).

### 3.4. Sociology and Diet

**Figure 6:**
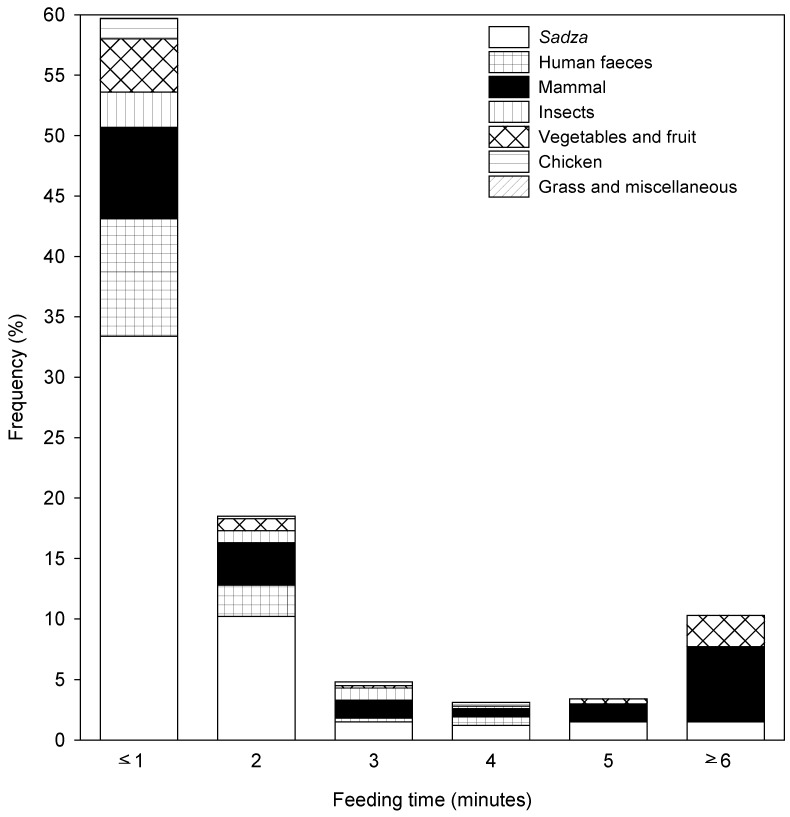
The duration of 689 observed dog meals, and the food items being fed on at each meal.

**Figure 7:**
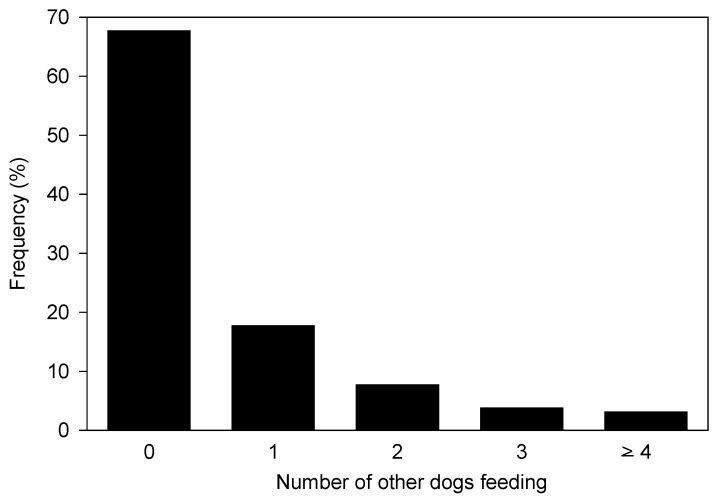
The numbers of other dogs feeding with the focal animal at each of the 689 meals.

The majority (59.9%) of observed meals were ≤1 min duration, and these largely consisted of sadza, human faeces, and mammal remains (Figure 6). Only 10.2% of meals lasted ≥6 min, and most of these involved extended feeding at carcasses or on gluts of insects. For the majority (67.7%) of meals, the focal animal was alone (Figure 7). 

## 4. Discussion

In common with the majority of free-ranging dog populations worldwide [5,7], dogs in the study area were predominantly scavengers of human waste: 88% of the dogs’ observed diet consisted of human-derived food, consisting of a wide range of vegetables, fruits, domestic animals, poultry, plus human faeces. With the exception of insects, all food items were inert and none were killed independently by the focal animals, although there were rare cases of predation by other dogs [9]. Furthermore, 87% of the observed diet was gained independently, and only 13% was deliberately fed by people. This is largely a consequence of the waste disposal practices of the people in the study area. Leftover sadza and other human foods such as cooked vegetables and fruit were usually thrown into pits on the homestead perimeter and were thus easily accessible to dogs. Furthermore, 59% of domestic animal fatalities were left in situ and provided freely accessible dog food.

Human faeces was also readily available because people typically defaecated in the open away from the homestead. Along with carcasses, dogs easily located this food source in the hinterland around homesteads by smell. Human faeces occurred in 56.2% of scats and contributed 20.5% by mass to the observed diet, ranking faeces as the fourth and third most important food item recorded, respectively.

Previous studies have recorded free-ranging dogs exhibiting coprophagy in India [13,28] and Ethiopia [14], but none have quantified the nutritional value of human faeces to the diet, or its supply. Nutritional analyses indicated that fresh human faeces was surprisingly nutritious, yielding 18.7% crude protein and 18.7 KJ/kg gross energy, which exceeded the results for sadza, the human staple (8.3% and 18.5 KJ/kg, respectively). Relative to mammal remains, which was the second most important food item in scats (81.3%) and the most important by mass in the observed diet (48.8%), human faeces was comparable to the lower range for crude protein, but near the maximum for gross energy. In general, up to 75% of the organic component of human faeces consists of undigested fat, protein, fibre, and carbohydrates [29], explaining its nutritional and energetic value.

By comparison to sadza and human faeces, mammal remains occurred erratically in the diet, perhaps due to their stochastic supply as slaughter offal and mortalities caused by disease and predation [6]. Vegetables and fruit were highly seasonal in the diet, corresponding to their fruiting and harvesting months. Insects were also seasonal, peaking in occurrence during the wet season. These patterns suggest that dogs consume food types relative to their availability. Hence the consistent occurrence of human faeces and sadza indicates that they are regularly available throughout the year. Based on estimates of faeces production in other communal lands [22], we calculate that with 92% of households not possessing a latrine or toilet, over 18 months there was potentially 64,800 kg available for consumption by dogs at a density of 1964 kg/km^2^, and at a rate of 109 kg/km^2^/month. This density is almost three times that for carcass remains in the GCL study area (696 kg/km^2^), and four times the density in the adjacent SWRA (484 kg/km^2^). Clearly, even based on these crude calculations, human faeces provides a consistent and substantial source of protein and energy-rich food for dogs. The value of this food source is augmented by its inert nature, requiring little effort to secure it, and its ease of location by smell.

The study area’s dog population was highly skewed towards juveniles, indicating high mortality rates. This mirrored the national communal land dog population, where 41% of dogs were aged ≤1 year, with 72% mortality in the first year and a mean life expectancy of 1.1 years [19]. Despite this high mortality rate, the dog population was estimated to be growing at 6.52%/annum [19]. One suggested explanation is that the consistent quantity and quality of human-derived food enables adult females to reproduce successfully throughout the year, offsetting the high mortality rate [19]. Dog population growth is probably also maintained by an expanding food supply, since in 1992 the national human population was growing at 3.13%/annum [19].

Most (64.2%) females were in good condition (scores 5 or 4) every month, and adult males fared even better, with 74% in these categories. The slight difference in condition should be expected from the physiological demands of pregnancy and lactation, which are up to four times greater than maintenance levels [30,31]. We did not collect detailed information on the nutritional status of the observed adult females, and its influence on their fertility and population demographics. However, the consistently good condition scores of females suggest that the quantity and quality of their food supply may indeed be sufficient to enable high fertility rates, and human faeces makes an important contribution.

The characteristics of the dogs’ food supply could also have influenced their social ecology. The majority (59.9%) of meals were small and consumed in less than 1 min, and largely consisted of sadza, human faeces, and mammal remains. For a larger majority of meals (67.7%) the feeding dog was alone. This suggests that the inert, dispersed, freely accessible, and small size of most food items did not necessitate cooperative foraging, as also suggested for free-ranging dogs in India [16]. This solitary behaviour is consistent with a plentiful food supply, which obviates the need for group behaviour or territoriality. Although domestic dogs are subject to artificial selection pressures which may modify their social ecology [15], the commensal relationship between humans and their ‘indigenous’ dogs in remote areas of Africa such as the GCL has probably remained unchanged for millennia [6,9,18]. Hence our study illustrates the conditions that may have encouraged wolves to abandon conspecific group behaviour, due to the availability of inert human waste, elucidating the self-domestication hypothesis [17]. 

The analysis of the dog diet was complicated by the need to quantify scavenged versus potentially predated food, human-fed versus independently-gained items, and the contribution of human faeces. To tackle this we used focal animal observations to estimate the mass of food ingested, which confirmed that scat analysis over-estimated the importance of small items. Scat analysis may also have mis-represented the occurrence of human faeces in the diet, due to our assumption that faeces was present only if sadza and relish remains occurred simultaneously in a scat. It is feasible that these items were directly eaten independently of each other by the dog, resulting in an over-representation of faeces; conversely, faeces may have been consumed that had contained only one or other food item, resulting in an under-representation. Nonetheless, this error was consistent throughout the study, and therefore the a-seasonal pattern of human faeces occurrence in the diet was credible. 

Scat analysis showed a wider range of food items than found in the observed diet. For example, 18 wild mammal species were found in scats compared to only five in the observed diet. A possible explanation is that some owners hunted small game such as springhares with dogs, and encroached into the SWRA with and without dogs to set snares [9], but with the advanced notice of our observation sessions they may have avoided hunting. Hence the scat analysis may give a more accurate quantification of the range of food items eaten by dogs and their owners, while observations provide a better assessment of ingested diet, suggesting that both methods are complementary and necessary when studying free-ranging dogs.

The substantial anthropogenic subsidy to the diet of the study area’s dog population was partly due to the lack of latrines and toilets; only 8% of households possessed one or the other. In a national survey of Zimbabwean communal lands, ownership of sanitation facilities ranged from 10% to 91% of households, with the lowest percentages in remote and under-developed communities [19]. The contribution of human faeces to dog diet is thus likely to recede in areas experiencing greater socio-economic development. However, this seems unlikely to limit the dog population, given that adult dog condition remains good in communal lands with higher levels of development, indicating a still plentiful and high quality food supply [18]. In addition, an increased production of edible waste would likely offset the reduced availability of human faeces in more developed areas. Ultimately, a transition may occur when socio-economic development improves the Dog Development Index, enabling owners to increase their investment in dog health and reproductive management, resulting in lower fertility rates [32].

The context of our study and its results are probably typical of many rural areas of developing countries where the Dog Development Index is low. In these situations, dogs pose varied threats to wildlife conservation and human health. For example, in Zimbabwean communal lands, dogs compete with vultures for carcasses [6,7] and act as reservoirs and vectors of canid disease to humans and wildlife [8,9,33]. However, as illustrated by this study, in these contexts they also provide an important but undervalued service as waste recyclers [34]. By consuming human faeces, dogs remove potentially harmful bacteria and excrete viruses in their own faeces that maintain immunity amongst local people [35]. This illustrates the inherent benefits and costs of free-ranging and feral dogs [5,36,37]. Nonetheless, improved sanitation seems unlikely to reduce fertility of free-ranging dogs, because they can switch opportunistically to other anthropogenic food subsidies. Instead, effective vaccination campaigns and reproductive control remain the most appropriate tools for addressing problems associated with free-ranging dog populations [10].

## Figures and Tables

**Table 1:** Frequency of occurrence (%F) of the principle food items in 945 dog scats, compared to the relative mass of items from 689 observed meals, presented as relative frequency of occurrence (%RF).

Food Item	Scats (%F)	Observed (%RF)
Sadza ^1^	87.9	22.1
Mammal meat, bones and skin ^2^	81.3	48.8
Vegetables and fruit ^3^	69.8	2.3
Human faeces	56.2	20.5
Grass	24.9	0.1
Insects ^4^	11.7	3.1
Chicken ^5^	6.7	1.8

^1^ Maize *Zea mays*, millet *Pennisetum americanum*, sorghum *Sorghum bicolor*, rapoko *Eleusine coracana*. ^2^ Goat, springhare, cow, sheep, dog, impala *Aepyceros melampus*, warthog *Phacochoerus aethiopicus*, donkey, cat, baboon *Papio ursinus*, bush squirrel *Paraxerus cepapi*, duiker *Sylvicapra grimmia*, kudu *Tragelaphus strepsiceros*, genet *Genetta tigrina* and *G. feline*, white-tailed mongoose *Ichneumia albicauda*, slender mongoose *Herpestes sanguineus*, scrub hare *Lepus saxitilis*, mouse *Tatera* spp. and *Saccostomus* spp., buffalo *Syncerus caffer*, striped polecat *Ictonyx striatus*, side-striped jackal *Canis adustus*, Sharpe’s grysbok *Raphicerus sharpei*, bushbuck *Tragelaphus scriptus*, African elephant *Loxodonta africana*, leopard *Panthera pardus*. ^3^ Cowpeas *Vigna unguiculata*, pumpkin *Cucurbita* spp., cucumber *Cucumis* spp., sweet potato *Ipomoea batatas*, tomato *Lycopersicon esculeutum*, onion *Aleum cepa*, rape *Brassica* spp., okra *Abelmoschus esculentus*, groundnuts *Voandzeia* spp., watermelon *Citrullus lanatus*, *Diospyros mespiliformis* fruit, *Berchemia discolor* fruit, *Grewia flavescens* fruit, *Ampelociccus* spp. fruit. ^4^ Grasshoppers *Acridida* spp., locusts *Locusta* spp., hawk moths (adult and larvae), chafer beetles *Rutelidida* spp., termites, dung beetles *Scarabaeida* spp., crickets *Gryllida* spp. ^5^ Bones, feathers, and eggshell.

**Table 2:** The mean crude protein and gross energy content of human faeces and sadza, compared with ranges for the mammalian remains, insects, and vegetable and fruit species eaten by dogs (see Table 1) taken from studies of food in Zimbabwe [27].

Food Item	Crude Protein (%)	Gross Energy (MJ/kg)
Human faeces	18.7	18.7
Sadza	8.3	18.5
Mammal meat, bones and skin	13.5–30.1	3.5–18.8
Insects	12.9–15.1	5.0–5.9
Vegetables and fruit	0.5–27.0	0.6–24.7
Chicken	10.0–26.7	8.2–14.6

## Supplementary Materials

The following are available online at http://www.mdpi.com/2076-2615/8/5/67/s1, Dog condition score.

## References

[1] Hughes J., Macdonald D.W. (2013). A review of the interactions between free-roaming domestic dogs and wildlife. Biol. Conserv..

[2] Butler J.R.A., Linnell J.D.C., Morrant D., Athreya V., Lescureux N., McKeown A., Gompper M.E. (2014). Dog eat dog, cat eat dog: Social-ecological dimensions of dog predation by wild carnivores. Free-Ranging Dogs and Wildlife Conservation.

[3] WHO (2005). World Health Organization Expert Consultation on Rabies.

[4] Smout F.A., Skerratt L.F., Butler J.R.A., Johnson C.N., Congdon B.C., Thompson R.C.A. (2017). The hookworm *Ancylostoma ceylanicum*: An emerging public health risk in Australian tropical rainforests and Indigenous communities. One Health.

[5] Gompper M.E., Gompper M.E. (2014). The dog-human-wildlife interface: Assessing the scope of the problem. Free-Ranging Dogs and Wildlife Conservation.

[6] Butler J.R.A., du Toit J.T. (2004). Diet of free-ranging domestic dogs (*Canis familiaris*) in rural Zimbabwe: Implications for wild scavengers on the periphery of wildlife reserves. Anim. Conserv..

[7] Vanak A.T., Dickman C.R., Silva-Rodriguez E.A., Butler J.R.A., Ritchie E.G., Gompper M.E. (2014). Top-dogs and under-dogs: Competition between dogs and sympatric carnivores. Free-Ranging Dogs and Wildlife Conservation.

[8] Knobel D.L., Butler J.R.A., Lembo T., Critchlow R., Gompper M.E., Gompper M.E. (2014). Dogs, disease, and wildlife. Free-Ranging Dogs and Wildlife Conservation.

[9] Butler J.R.A., du Toit J.T., Bingham J. (2004). Free-ranging domestic dogs *Canis familiaris* as predators and prey in rural Zimbabwe: Threats of competition and disease to large wild carnivores. Biol. Conserv..

[10] Taylor L.H., Wallace R.M., Balaram D., Lindenmayer J.M., Eckery D.C., Mutonono-Watkiss B., Parravani E., Nel L.H. (2017). The role of dog population management in rabies elimination—A review of current approaches and future opportunities. Front. Vet. Sci..

[11] Knobel D.L., Cleaveland S., Coleman P.G., Fevre E.M., Meltzer M.I., Miranda M.E., Shaw A., Zinsstag J., Meslin F.X. (2005). Re-evaluating the burden of rabies in Africa and Asia. Bull. World Health Organ..

[12] Ogada D.L., Keesing F., Virani M.Z. (2012). Dropping dead: Causes and consequences of vulture population declines worldwide. Ann. N. Y. Acad. Sci. USA.

[13] Vanak A.T., Gompper M.E. (2009). Dietary niche separation between sympatric free-ranging domestic dogs and Indian foxes in central India. J. Mammal..

[14] Atickem A., Bekele A., Williams S.D. (2010). Competition between domestic dogs and Ethiopian wolf (*Canis simensis*) in the Bale Mountains National Park, Ethiopia. Afr. J. Ecol..

[15] Boitani L., Ciucci P., Ortolani A., Jensen P. (2007). Behaviour and social ecology of free-ranging dogs. Behavioral Biology of Dogs.

[16] Sen Majumder S., Bhadra A., Ghosh A., Mitra S., Bhattacharjee D., Chatterjee J., Nandi A.K., Bhadra A. (2014). To be or not to be social: Foraging associations of free-ranging dogs in an urban ecosystem. Acta Ethol..

[17] Marshall-Pescini S., Cafazzo S., Viranyi Z., Range F. (2017). Integrating social ecology in explanations of wolf–dog behavioral differences. Curr. Opin. Behav. Sci..

[18] Butler J.R.A. (1998). The Ecology of Domestic Dogs Canis familiaris in the Communal Lands of Zimbabwe. Ph.D. Thesis.

[19] Butler J.R.A., Bingham J. (2000). Demography and dog-human relationships of the dog population in Zimbabwean communal lands. Vet. Rec..

[20] Butler J.R.A. (2000). The economic costs of wildlife predation on livestock in Gokwe communal land, Zimbabwe. Afr. J. Ecol..

[21] Brooks R. (1990). Survey of the dog population of Zimbabwe and its level of rabies vaccination. Vet. Rec..

[22] Bradley M. (1995). Personal communication.

[23] Henschel J.R., Skinner J.D. (1990). The diet of spotted hyaenas *Crocuta crocuta* in Kruger National Park. Afr. J. Ecol..

[24] Sillero-Zubiri C., Gottelli D. (1995). Diet and feeding behavior of Ethiopian wolves (*Canis simensis*). J. Mammal..

[25] Corbett L.K. (1989). Assessing the diet of dingoes from faeces: A comparison of 3 methods. J. Wildl. Manag..

[26] Riney T. (1960). A field technique for assessing physical condition of some ungulates. J. Wildl. Manag..

[27] Chitsiku I.C. (1989). Nutritive value of foods of Zimbabwe. Zambezia.

[28] Oppenheimer E.C., Oppenheimer J.R. (1975). Certain behavioral features in the pariah dog (*Canis familiaris*) in West Bengal. Appl. Anim. Ethol..

[29] Rose C., Parker A., Jefferson B., Cartmell E. (2015). The characterization of feces and urine: A review of the literature to inform advanced treatment technology. Crit. Rev. Environ. Sci. Technol..

[30] Scantlebury M., Butterwisk R., Speakman J.R. (2000). Energetics of lactation in domestic dog (*Canis familiaris*) breeds of two sizes. Comp. Biochem. Physiol. Part A.

[31] Ontko J., Phillips P.H. (1958). Reproduction and lactation studies with bitches fed semi-purified diets. J. Nutr..

[32] Fung H.L., Calzada J., Saldana A., Santamaria A.M., Pineda V., Gonzalez K., Chaves L.F., Garner B., Gottdenker N. (2014). Domestic dog health worsens with socio-economic deprivation of their home communities. Acta Trop..

[33] McRee A., Wilkes R.P., Dawson J., Parry R., Foggin C., Adams H., Odoi A., Kennedy M.A. (2014). Serological detection of infection with canine distemper virus, canine parvovirus and canine adenovirus in communal dogs from Zimbabwe. J. S. Afr. Vet. Assoc..

[34] Coppinger R., Coppinger L. (2001). Dogs: A New Understanding of Canine Origin, Behavior and Evolution.

[35] Graves I.L., Oppenheimer J.R. (1975). Human viruses in animals in West Bengal: An ecological analysis. Hum. Ecol..

[36] Morrant D.S., Wurster C., Johnson C.N., Butler J.R.A., Congdon B.C. (2017). Prey use by dingoes in a contested landscape: Ecosystem service provider or biodiversity threat?. Ecol. Evol..

[37] Morrant D.S., Johnson C.N., Butler J.R.A., Congdon B.C. (2017). Biodiversity friend or foe: Ecology of a top predator, the dingo in contested landscapes of the Australian Wet Tropics. Austral Ecol..

