# Supplementary material for: Anthropogenic Food Subsidy to a Commensal Carnivore: The Value and Supply of Human Faeces in the Diet of Free-Ranging Dogs
Source: Animals (Basel). 2018 Apr 27;8(5):67. doi: 10.3390/ani8050067 (PMC5981278; doi:10.3390/ani8050067)

**Supplementary Material**

**Dog condition score**

A qualitative condition score was awarded monthly to every focal animal and other associated adult animals sighted. The score used a five-point scale based on the visibility of skeletal features under the skin, where 5 represented very good condition and 1 represented very poor. The photographs below illustrate the five-point scale.

Condition score 5: very good


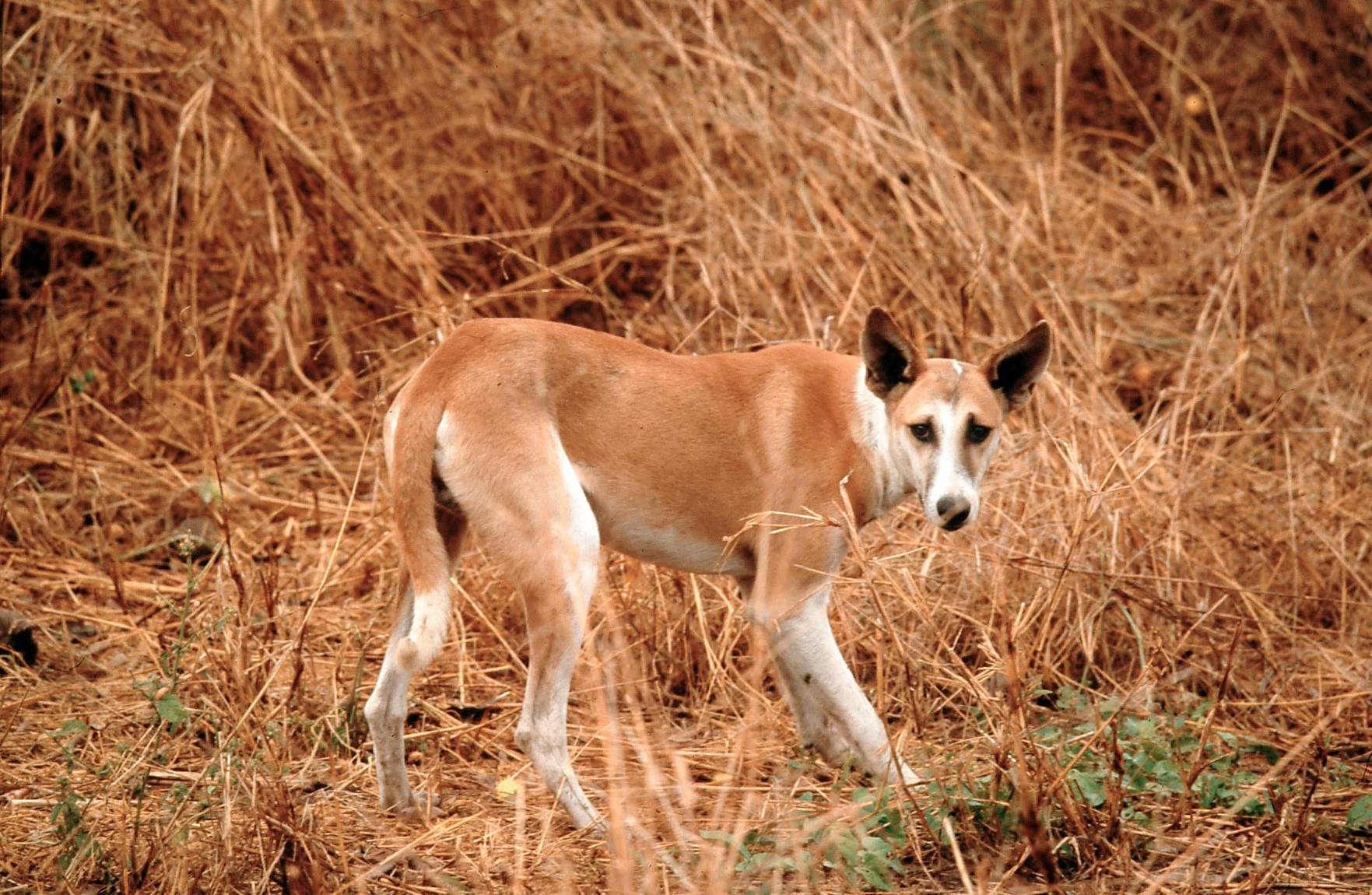


Condition score 4
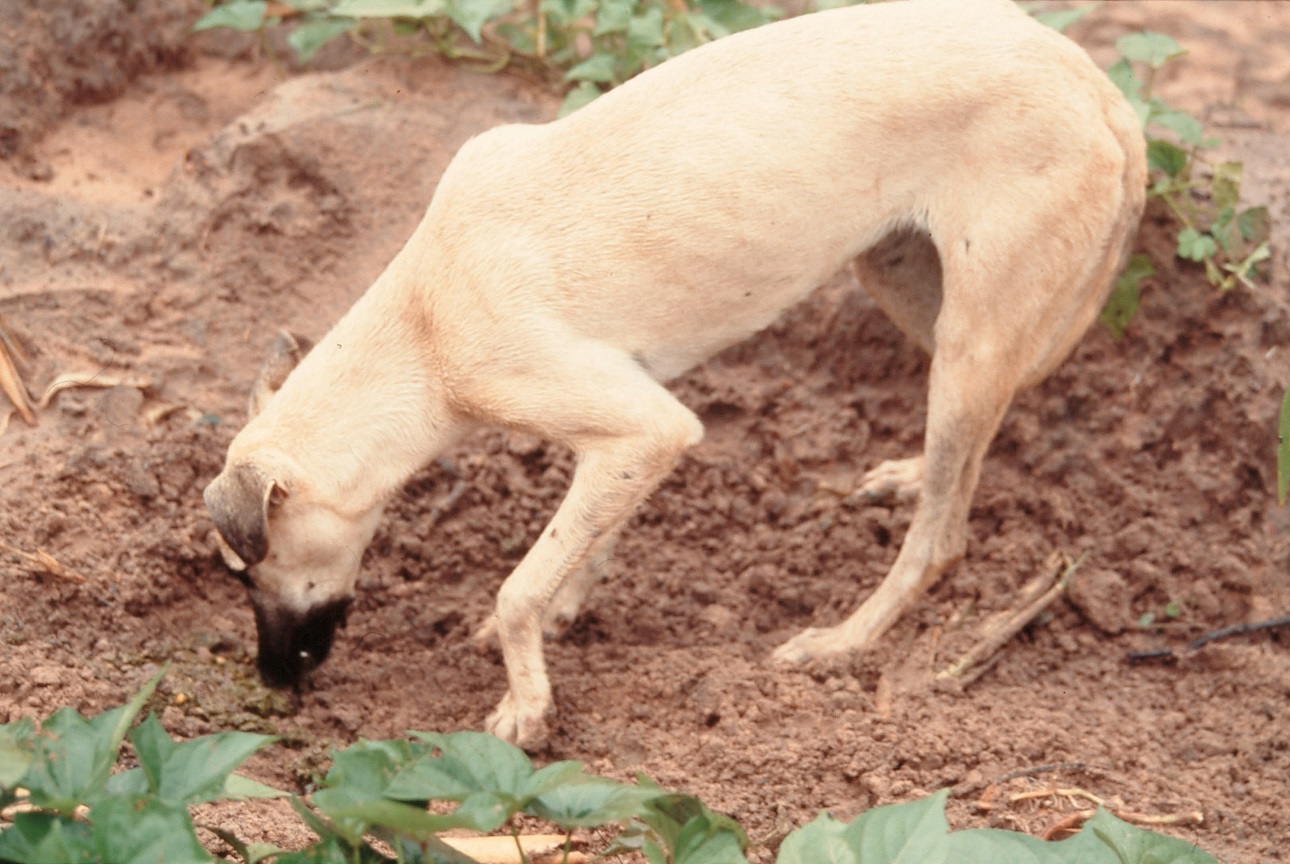


Condition score 3


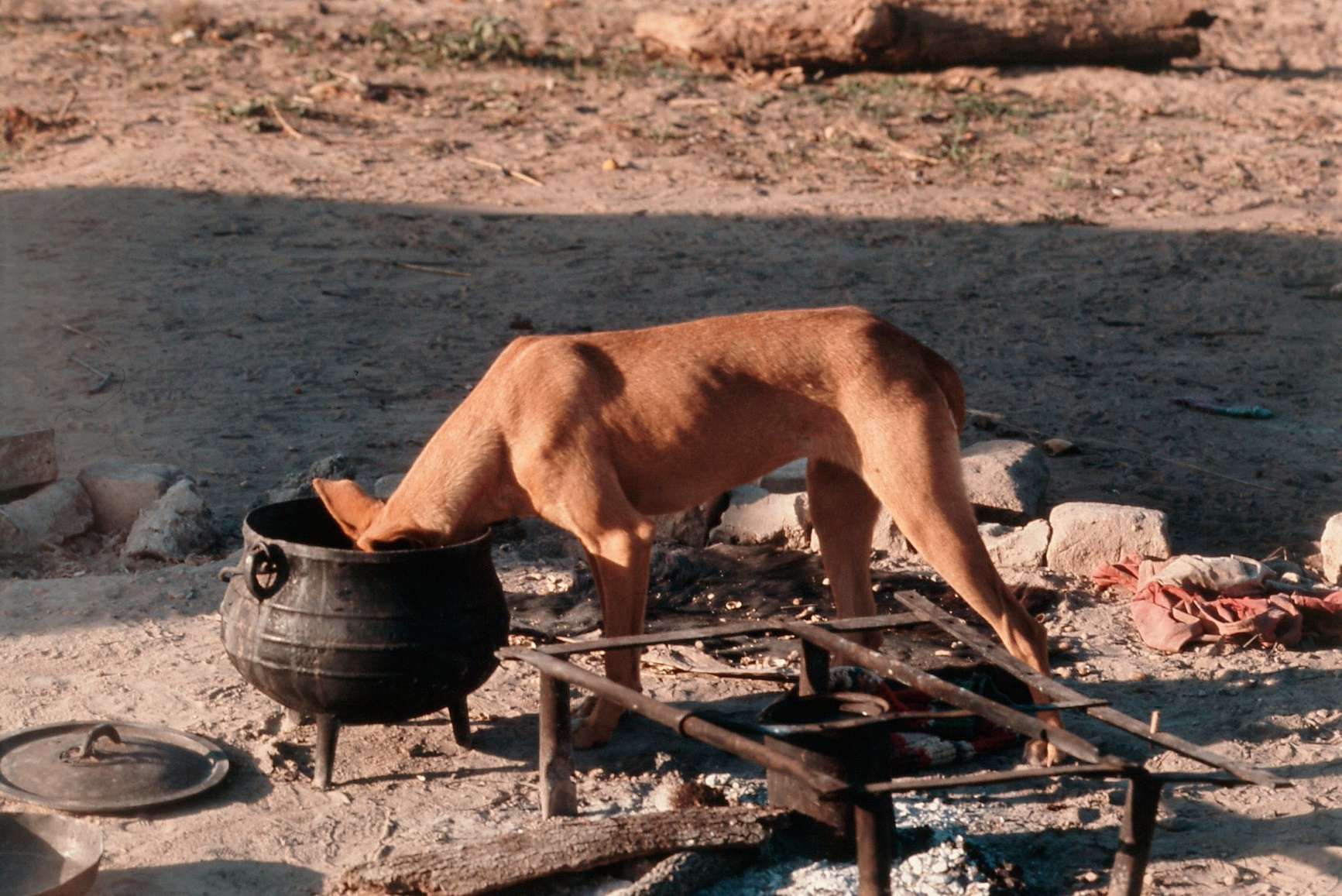


Condition score 2


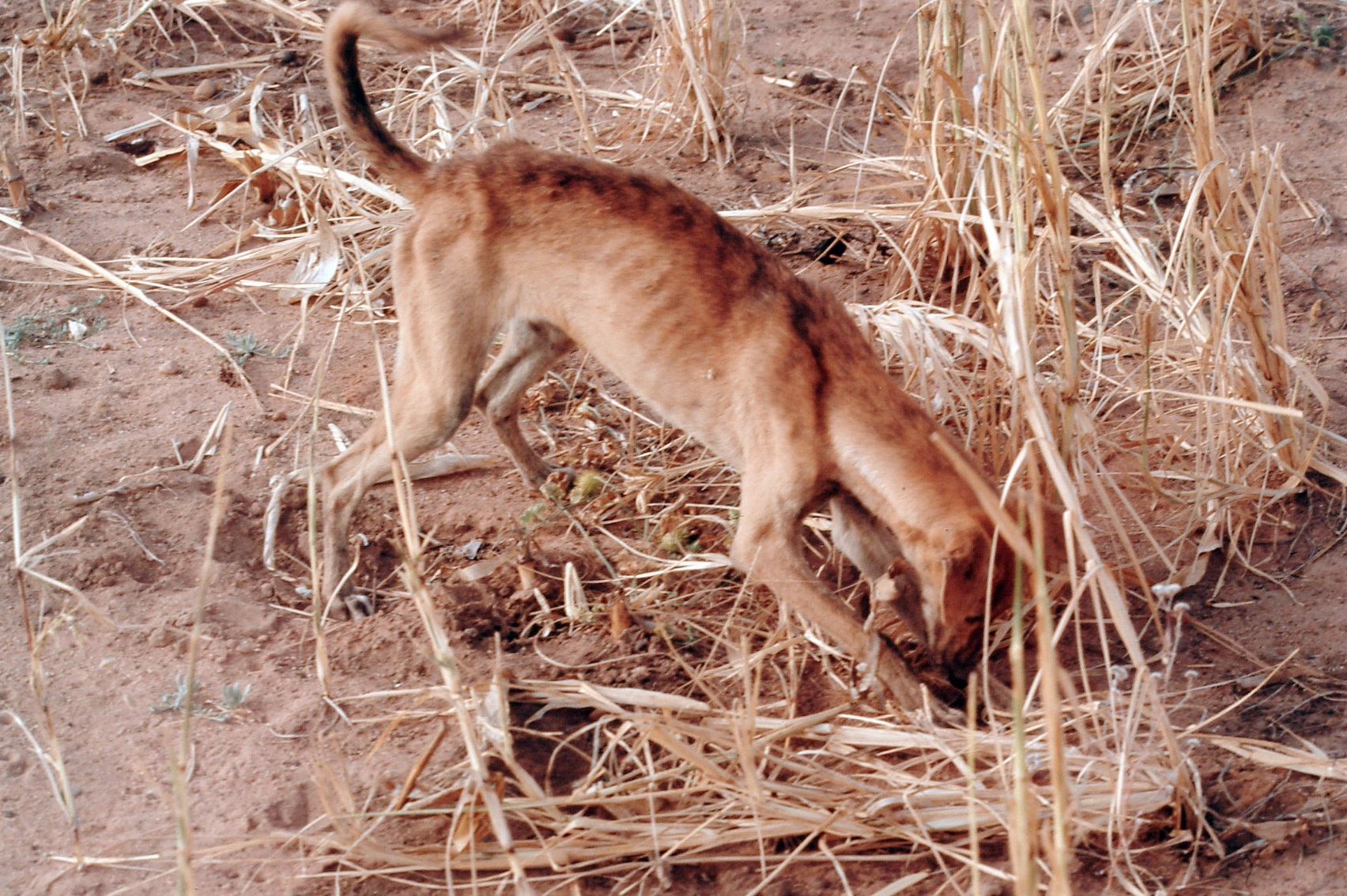


Condition score 1: very poor


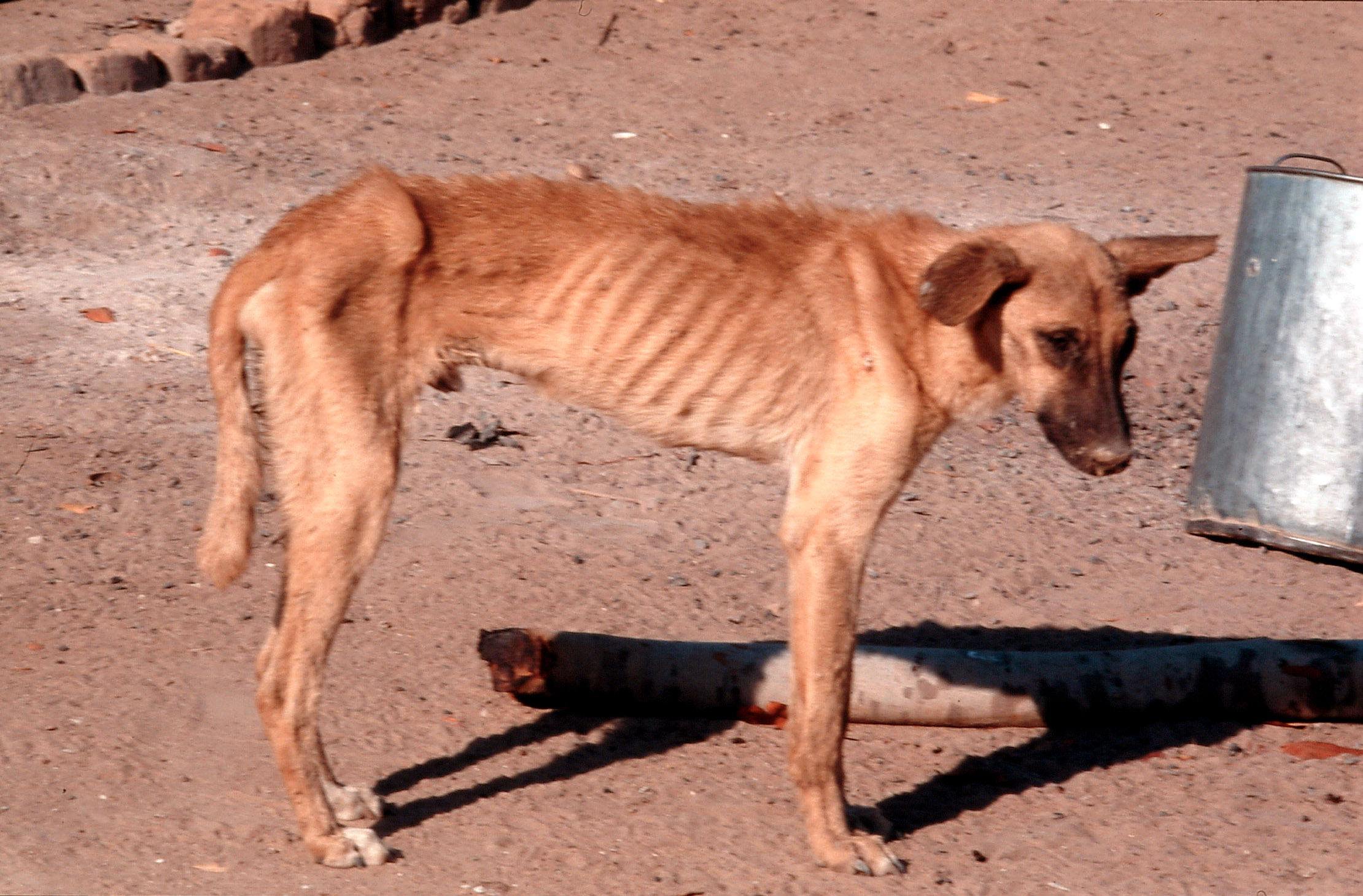

Supplement: Supplementary file 1 [file animals-08-00067-s001.zip › animals-290124-supplementary.docx]
